# Hypoxia directed migration of human naïve monocytes is associated with an attenuation of cytokine release: indications for a key role of CCL26

**DOI:** 10.1186/s12967-020-02567-7

**Published:** 2020-10-21

**Authors:** Lars Hummitzsch, Rouven Berndt, Matthias Kott, Rene Rusch, Fred Faendrich, Matthias Gruenewald, Markus Steinfath, Martin Albrecht, Karina Zitta

**Affiliations:** 1 Department of Anesthesiology and Intensive Care Medicine, UKSH, Schwanenweg 21, 24105 Kiel, Germany; 2 Clinic for Applied Cellular Medicine, UKSH, Kiel, Germany; 3 Department of Cardiovascular Surgery, UKSH, Kiel, Germany

**Keywords:** Monocytes, Hypoxia, Migration, Cytokines, CCL26

## Abstract

**Background:**

Numerous tissue-derived factors have been postulated to be involved in tissue migration of circulating monocytes. The aim of this study was to evaluate whether a defined hypoxic gradient can induce directed migration of naïve human monocytes and to identify responsible autocrine/paracrine factors.

**Methods:**

Monocytes were isolated from peripheral blood mononuclear cells, transferred into chemotaxis chambers and subjected to a defined oxygen gradient with or without the addition of CCL26. Cell migration was recorded and secretome analyses were performed.

**Results:**

Cell migration recordings revealed directed migration of monocytes towards the source of hypoxia. Analysis of the monocyte secretome demonstrated a reduced secretion of 70% (19/27) of the analyzed cytokines under hypoxic conditions. The most down-regulated factors were CCL26 (− 99%), CCL1 (− 95%), CX3CL1 (− 95%), CCL17 (− 85%) and XCL1 (− 83%). Administration of recombinant CCL26 abolished the hypoxia-induced directed migration of human monocytes, while the addition of CCL26 under normoxic conditions resulted in a repulsion of monocytes from the source of CCL26.

**Conclusions:**

Hypoxia induces directed migration of human monocytes in-vitro. Autocrine/paracrine released CCL26 is involved in the hypoxia-mediated monocyte migration and may represent a target molecule for the modulation of monocyte migration in-vivo.

## Introduction

Peripheral blood monocytes representing about 4–8% of all circulating leukocytes, possess only a limited life span in the blood circulation and undergo spontaneous apoptosis after 24–48 h [1]. However, upon adequate stimuli, monocytes interrupt their apoptotic program and migrate into peripheral tissues where they can differentiate into macrophages and dendritic cells and exhibit a wide range of different functions including phagocytosis of microbes and cell debris, antigen presentation, processing of extracellular matrix, secretion of cytokines, chemokines, growth factors, reactive oxygen species, and complement components [2–4].

Various pathological conditions like bacterial infections, ischemic diseases (e.g. myocardial and cerebral infarction), neoplasia, chronic inflammation, and autoimmune diseases (e.g. rheumatic arthritis) are typically associated with the tissue infiltration of circulating monocytes [5, 6]. There is evidence that the proinflammatory immune response within the diseased tissue shifts the balance of oxygen supply towards tissue hypoxia, promoting the formation of a specific inflammatory microenvironment, which is characterized by high concentrations of pro-inflammatory cytokines/chemokines and low levels of oxygen [7]. As a consequence, additional monocytes may migrate along a specific chemotactic gradient and accumulate within the hypoxic areas of the affected tissues [3]. The infiltration of monocytes and the release of inflammatory mediators both amplify the pro-inflammatory immune response and can lead to an expansion of the initial tissue injury [8]. A number of tissue derived factors, such as chemokines, complement factors as well as products of tissue matrix degradation have been postulated to be responsible for the directed migration of monocytes during inflammatory processes [4]. While MCP-1, MIP-1α, CCL5, MCP-3, and CCL7 are commonly reported to be potent chemokines recruiting monocytes/macrophages into inflammatory lesions, there is also a great number of other less described chemokines (e.g. CCL4, CCL8, CCL13, CCL14, CX3CL1, and CCL26) that are believed to be involved in the regulation of monocyte migration [9, 10].

As tissue inflammation is associated with hypoxia [11], several authors proposed that the monocyte-macrophage system responds directly to changes in oxygen supply and that the tissue oxygen gradient may be a critical factor for monocyte migration into inflammatory regions [3, 11, 12]. However, due to the lack of suitable in-vivo and in-vitro models, it has not been clarified yet, whether hypoxia alone can recruit monocytes or if the hypoxia-induced secretion of cytokines by other cell types (e.g. endothelial cells, fibroblasts) is crucial for the directed migration of monocytes towards hypoxic areas. In the current study, we have therefore established a migration chamber model in combination with defined oxygen gradients to investigate whether hypoxia alone can induce directed migration of naïve human monocytes and which secreted factor(s) are involved. To the best of our knowledge, our study is the first one showing that a defined gradient of hypoxia alone is sufficient to induce directed migration of human monocytes. A deeper understanding of the underlying mechanisms of monocyte migration into inflamed and hypoxic areas could lead to the development of novel treatment strategies for several diseases associated with the monocyte infiltration (e.g. rheumatic arthritis, atherosclerosis, myocardial infarction, and neoplasia).

## Methods

### Ethics and monocyte isolation

The study was approved by the local Ethics Committee of the University Medical Center Schleswig–Holstein, Kiel, Germany (protocol identification: D519/18). Peripheral blood monocytes were obtained from leukapheresis products from the Department of Transfusion Medicine (University Hospital Schleswig Holstein, Kiel, Germany) and isolated by Ficoll-Paque PLUS (GE Healthcare, Chicago, USA) density gradient centrifugation and selective adherence to cell culture surfaces according to an established protocol [13].

### Migration chambers and induction of oxygen gradients

**Fig. 1:**
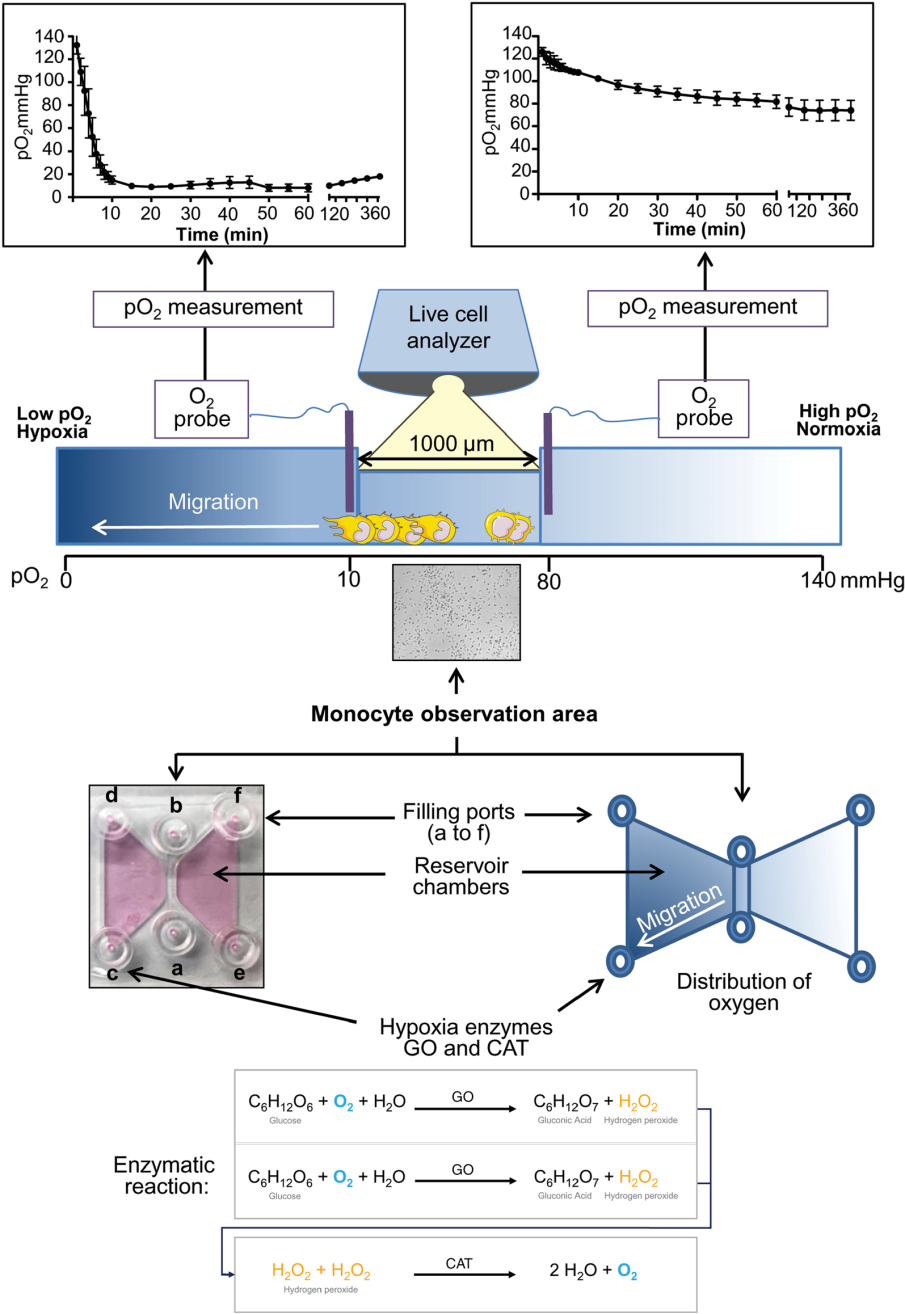
Experimental setup and use of the hypoxia migration chamber. pO_2,_ partial pressure of oxygen; GO, glucose-oxidase; CAT, catalase

The hypoxia system employed in our study represents an easy to handle application, which also can be utilized by other researchers for the study of migratory behavior of various cell types under defined oxygen gradients. For the described experiments a µ-slide migration chamber system (Ibidi, Martinsried, Germany) was applied. The chamber includes a 1 mm wide observation area, where cells are seeded and cellular movements can be recorded. The observation area is connected with reservoir chambers enabling the formation of chemical gradients by the addition of chemotactic substances into the respective filling ports (Fig. 1).

Based on the diffusion characteristics of the applied substance throughout the chamber, cells located inside the observation area become exposed to different concentrations of the administered chemoattractant. To establish stable oxygen gradients within this system, the migration chamber was combined with our previously described two-enzyme hypoxia system with minor modifications [14, 15]. Monocytes were cultured in RPMI-1640 culture medium (Lonza, Cologne, Germany) containing 1 g/l glucose and supplemented with 10% human AB-serum (Center for Clinical Transfusion Medicine, Tuebingen, Germany). Initially, the migration chamber was filled with culture medium according to the manufacturer’s protocol. Several test series were performed to identify the optimal enzyme concentration for the generation of a stable and physiologically relevant oxygen gradient within the migration chamber (data not shown). Based on the respective results, 15 µl RPMI-1640 cell culture medium containing 600 U/ml glucose-oxidase (GO; Sigma-Aldrich, Schnelldorf, Germany) and 36,000 U/ml catalase (CAT; Sigma-Aldrich) was added to the filling port c of the respective reservoir chamber. As controls, 15 µl RPMI-1640 cell culture medium without enzymes or medium containing heat-inactivated enzymes was added to the filling port of the other reservoir chamber. To determine the temporal and spatial formation of an oxygen gradient within the chamber, partial pressure of oxygen (pO_2_) and its temporal decline after the addition of GO and CAT was measured by using a flexible pO_2_ probe (LICOX^®^ CMP Oxygen Catheter, Integra, Plainsboro, NJ), that was placed in different positions throughout the chamber (Fig. 1).

### Cell migration assays

Cell migration studies were performed using the µ-slide migration chamber system (Ibidi) according to the manufacturer’s protocol. Initially, 6 µl of cell suspension (3.5 × 10^6^ cells/ml) were loaded into the observation area (ports a and b, Fig. 1). After 1 h, non-adherent cells (consisting of mainly lymphocytes) were removed by washing the observation channel carefully with 10 µl culture medium. After filling the lateral reservoirs of the chamber with 65 µl fresh culture medium, 15 µl of hypoxia enzyme-containing culture medium were added to the filling port c, while 15 µl of standard cell culture medium were placed into filling port e. Normoxic control experiments were performed by omitting the hypoxia inducing enzymes (GO and CAT) from the respective culture media. To exclude the possibility that the two proteins GO and CAT themselves (and not the resulting hypoxic gradient induced by GO and CAT) are responsible for the observed directed migration of monocytes, control experiments using heat inactivated hypoxia enzymes (GO and CAT) were performed. For this purpose, the final solutions of GO and CAT were heated to 95 °C (10 min) and cooled on ice (1 min). This procedure was repeated 5 times and the potential of the enzymes to induce hypoxia was evaluated. If the ability of the enzymes to induce hypoxia was abrogated, 15 µl of inactivated GO/CAT enzyme solution were transferred into the filling port c. Cellular migration was recorded for 5 h using a live cell analyzer (JuLiBr, NanoEntek, Seoul, Korea) with the time-lapse interval set to 2 min. Cellular tracking was performed by using the plugin "Manual Tracking" for the ImageJ software 1.41 (National Institutes of Health, NIH, Bethesda, USA). The migration behavior was then further analyzed with the ImageJ plugin "Chemotaxis and Migration Tool 2.0" (Ibidi) and the following parameters were generated to evaluate directed cell migration: The center of mass (COM), forward migration index (FMI), cell velocity (CV) and cell directness (CD). For statistical evaluation of the parameters, the Rayleigh test was employed [16]. All experiments were independently performed 3–5 times and an average of 30 cells was analyzed per experiment. To investigate the involvement of CCL26 in hypoxia-induced cell migration, experimental settings using recombinant human CCL26 (R&D Systems, Minneapolis, USA) were performed. Cells were seeded into the migration chamber as described above and 30 µl of RPMI-1640 culture media supplemented with CCL26 (1 µM) was added to the filling port c, while, 30 µl standard culture media was added to filling port e as control. In another setting, the migration chamber was evenly filled with cell culture medium supplemented with CCL26 (final concentration 0.3 µM) before the generation of a hypoxic gradient.

### Protein isolation and collection of conditioned media

To maximize the yield of protein and culture supernatants for subsequent biochemical analyses, additional hypoxia experiments were performed in parallel in 6-well plates employing our established hypoxia enzyme insert system with minor modifications [17]. As monocytes located inside the µ-slide migration chamber system (Ibidi) were exposed to hypoxic gradients representing oxygen levels between 10 and 80 mmHg (Fig. 1), we therefore decided to adjust the pO_2_ in the culture medium of 6-well plates to approximately 40 mmHg. Briefly, human monocytes were seeded into the 6-well plates at a density of 160,000 cells/cm^2^. After 1 h, non-adherent cells (mainly lymphocytes) were removed by exchanging the culture medium. Hypoxia was induced by placing the cell culture inserts equipped with a gas-permeable membrane (BioCrystal Ltd., Westerville, USA) into the respective well. Inserts were filled with DMEM-F12 culture medium containing 600 U/ml GO and 36,000 U/ml CAT, resulting in a rapid decrease of pO_2_ to levels of approximately 40 mmHg within the monocyte culture [pO_2_ to levels were confirmed by using a flexible pO_2_ probe (LICOX^®^ CMP Oxygen Catheter, Integra)]. Additionally, cells were cultured under hypoxic and normoxic conditions with CCL26 at a final concentration of 0.3 µM. After 1 and 5 h of hypoxia, the cell culture inserts were removed and monocyte supernatants were collected and stored at − 80 °C. For protein extraction cells were lysed using RIPA buffer containing 150 mM sodium chloride, 1% NP-40, 1%, sodium deoxycholate, 0.1% sodium dodecyl sulfate (SDS) and, 50 mM Tris–HCl (pH 7.6; all from Sigma-Aldrich). Cells were scraped off the bottom using a rubber policeman, disrupted with a syringe, and sonicated on ice for 10 min. The remaining solution was centrifuged 15 min at 14,000×*g* at 4 °C. Supernatants were frozen at − 20 °C until use.

### Cytokine/chemokine secretome analyses

Analyses of the cytokines and chemokines secreted by monocytes under hypoxic and normoxic conditions were performed using Proteome Profiler Human Chemokine Arrays (ARY017, R&D Systems) according to the manufacturer`s protocol provided with the assay kit. 1 ml of pooled cell culture supernatant derived from N = 5 independently performed experiments was applied to the respective array membranes. Expression levels of 31 cytokines/chemokines were simultaneously evaluated by densitometric analyses of the arrays using the ImageJ 1.41 software (NIH). For each spot on the membrane, the optical density was determined and the cut off signal level was set to 10% of the mean intensity of the respective reference spots. Based on these criteria regulation of 27/31 cytokines/chemokines could be analyzed and quantified while 4 cytokines/chemokines (CCL28, CXCL11, CXCL9 and CXCL17) showed an optical density below the cut off and were excluded.

### Western blot

Concentrations of protein samples were determined with Roti-Quant assays (Carl Roth, Karlsruhe, Germany). 20 μg of total protein were mixed with 4× Laemmli buffer (8% SDS, 40% glycerol, 20% 2-mercaptoethanol, 0.008% bromophenol blue, 0.25 M Tris–HCl, all from Sigma-Aldrich) and incubated for 3 min at 95 °C. Samples were separated by 8% SDS-PAGE and transferred to a PVDF membrane (Amersham Pharmacia Biotech, Piscataway, USA). After 2 h of blocking with TBST buffer containing 3% of bovine serum albumin (Carl Roth) at room temperature, the membranes were incubated overnight at 4 °C with specific primary antibodies, anti-CCL26 (1:100; R&D systems, Minneapolis, USA), and anti-RAGE (1:4000; Genetex, Irvine, USA). Signals from peroxidase-conjugated secondary antibodies (anti-rabbit, Cell Signaling Technology, 1:20,000) were detected using the ECL kit (ECL-Plus Western blotting Detection Reagents, Amersham Pharmacia Biotech). Protein bands on the membranes were visualized with the western blot imaging system Fusion FX (Vilber, Collégien, France) and intensities of the respective protein bands were analyzed using the ImageJ software 1.41 (NIH).

### Statistical analysis

All values are expressed as mean ± standard deviation (SD). Data of cell migration studies were analyzed by using circular statistics (Rayleigh test) available in the software "Chemotaxis and Migration Tool" (Ibidi). All other data were analyzed with the software Graph Pad Prism version 5.01 for Windows (GraphPad Software; San Diego, California, USA) and tested for normality using the Kolmogorov–Smirnov test. If normality was not present, data were transformed (arcsine of the square root of x) before using parametric tests. Statistical comparisons of two groups were performed using the Student’s t-test or One-sample t-test. A p-value < 0.05 was considered significant.

## Results

### Employing an enzymatic hypoxia model in combination with chemotaxis chambers stable oxygen gradients are obtained

The induction of oxygen gradients was performed by employing commercially available chemotaxis chambers (Ibidi) in combination with our recently described enzymatic hypoxia model [14, 15]. The addition of enzymes that induce hypoxia (GO and CAT) into the filling port of the chamber resulted in a rapid formation of an oxygen gradient throughout the whole migration chamber (Fig. 1). Measurements of pO_2_ at both sides of the observation area (Fig. 1, area between a and b) revealed that cells located inside the respective area are exposed to oxygen levels ranging from 10 to 80 mmHg (Fig. 1). Concerning the temporal component of hypoxia, measurements showed a rapid formation of oxygen gradients within the migration chamber (a drop in pO_2_ from 140 mmHg to levels below 10 mmHg was achieved in approximately 15 min). Hypoxic conditions were stable throughout the whole experiment lasting for at least 6 h (Fig. 1).

### Monocytes migrate towards the source of hypoxia

**Fig. 2:**
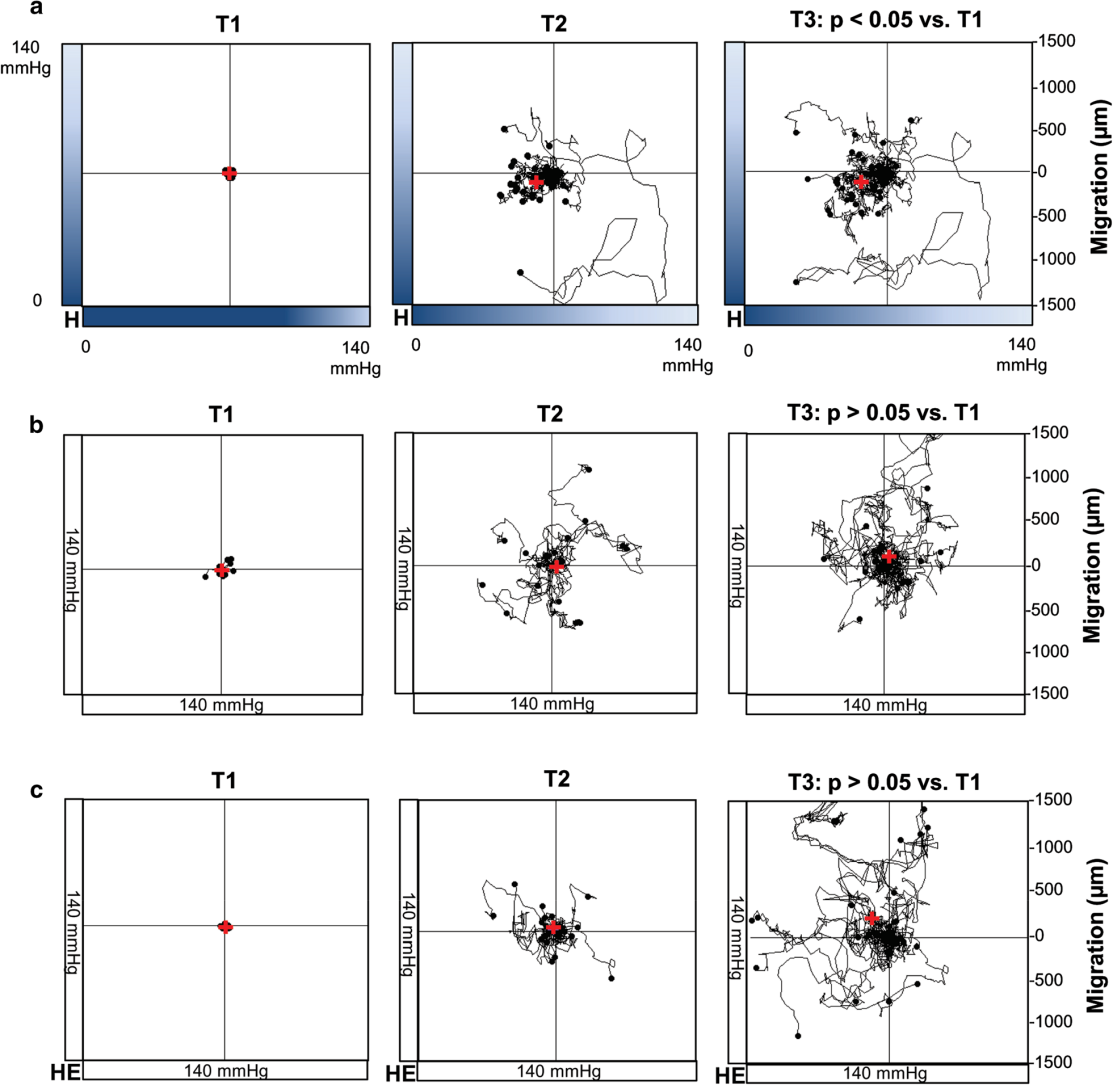
Effect of hypoxia on monocyte migration. The red cross represents the mean end point of the trajectory of cells subjected to an oxygen gradient (**a**), under normoxic conditions (**b**) or subjected to a gradient of heat-inactivated hypoxia-inducing enzymes (**c**). Each graph shows one representative experiment, containing migration data of at least N = 20 cells. T1, time point < 5 min; T2, time point = 2.5 h; T3, time point = 5 h; H, hypoxia; HE, heat inactivated enzymes (GO and CAT)

Monocytes seeded into the observation area of the migration chamber were subjected to oxygen gradients for 5 h. Recordings and analyses of monocyte movements revealed a statistically significant directed migration towards lower oxygen concentrations (hypoxia, p < 0.05, Fig. 2a). Normoxic conditions resulted in an erratic and non-directed movement of the monocytes (normoxia, p > 0.05, Fig. 2b). A graphical presentation of the outcome of each independently performed experiment is displayed in Additional file 1: Figure S1. Additional control experiments using heat-inactivated hypoxia enzymes (GO and CAT) also resulted in a non-directed movement of the monocytes (inactivated enzymes, p > 0.05, Fig. 2c), suggesting that the oxygen gradient and not a gradient of hypoxia generating enzymes GO and CAT themselves is responsible for the directed migration of human monocytes in-vitro.

### Hypoxia down regulates the secretion of various cytokines/chemokines by human monocytes

**Fig. 3:**
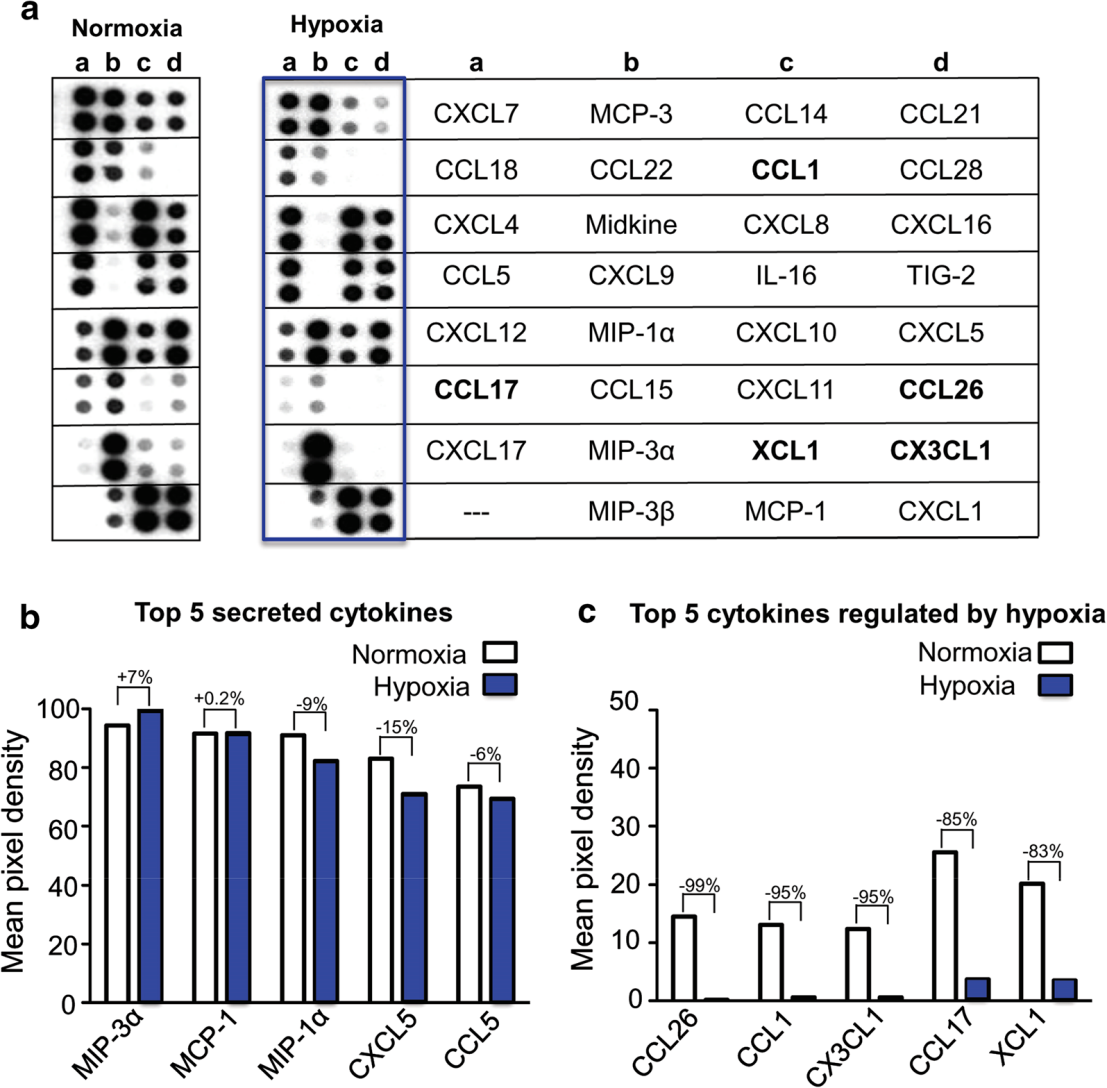
Proteome profiling arrays. **a** Representative array membranes detecting 31 cytokines involved in chemotaxis. The top 5 secreted cytokines are presented in **b** while the top 5 hypoxia regulated cytokines are shown in **c**. For a detailed description of the array proteins please refer to Additional file 1: Figure S2

To investigate whether the secretion of cytokines/chemokines from monocytes was also affected by hypoxia, proteome profiler arrays were performed with monocyte cell culture supernatants. The respective results show that hypoxia influences the secretion of various cytokines/chemokines. An overview of the secretome under normoxic and hypoxic conditions is shown in Fig. 3a. In total, a hypoxia-mediated reduced secretion of 70% (19/27) of the analyzed cytokines/chemokines was detected while 30% (8/27) of the cytokines/chemokines did not reveal any changes in their secretion pattern. The top 5 secreted proteins were: MIP-3α, MCP-1, MIP-1α, CXCL5, and CCL5 (Fig. 3b). The cytokines/chemokines with the strongest hypoxia-mediated reduction in secretion were: CCL26 (− 99%), CCL1 (− 95%), CX3CL1 (− 95%), CCL17 (− 85%) and XCL1 (− 83%) (Fig. 3c). A detailed description of the array proteins is shown in Additional file 1: Figure S2.

### CCL26 is involved in the hypoxia-induced migration of monocytes

**Fig. 4:**
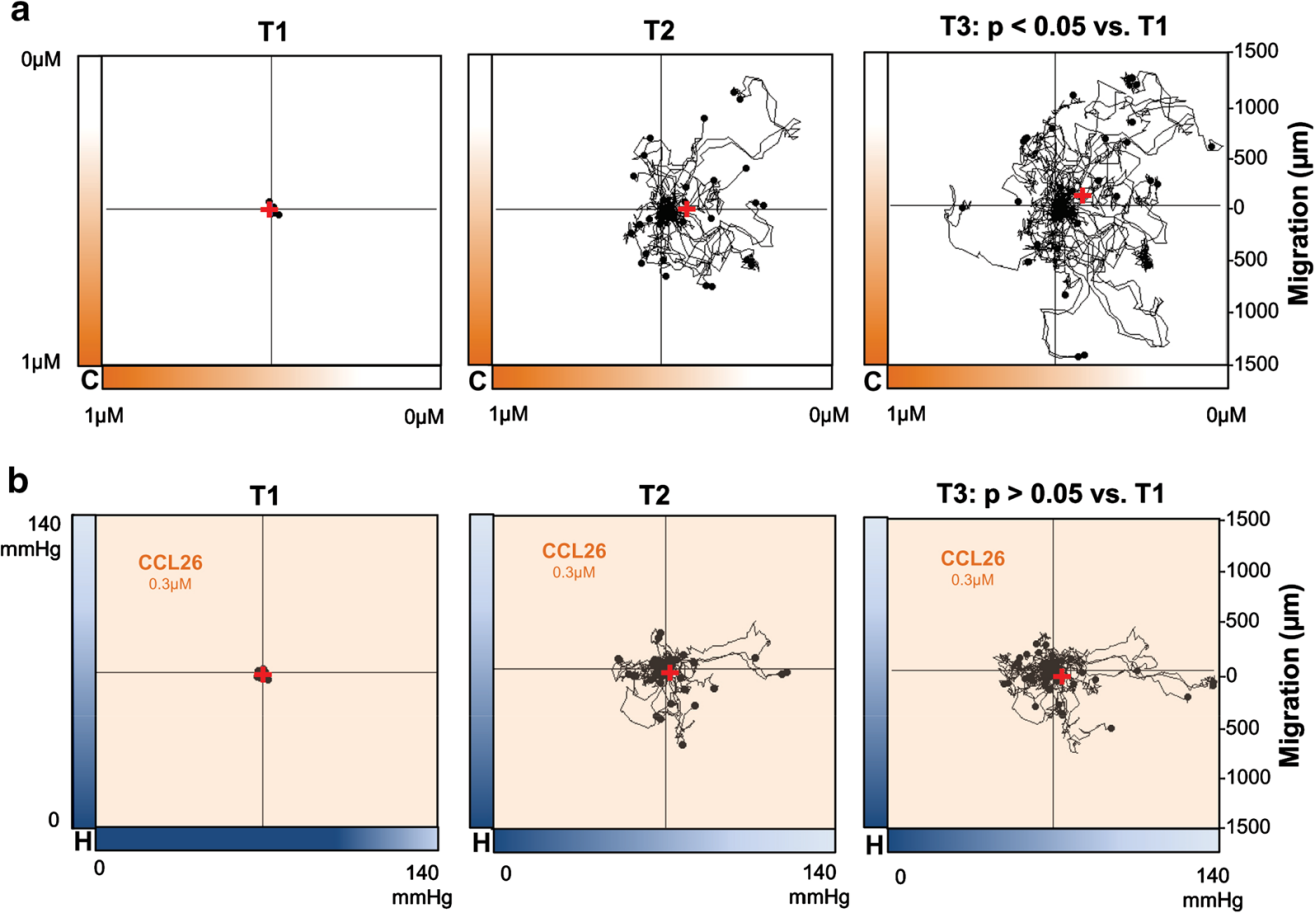
Effect of CCL26 on monocyte migration. The red cross in the graphics represents the mean end point of the trajectory of cells treated with a gradient of CCL26 under normoxic conditions without hypoxic gradient (**a**) or with an oxygen gradient in combination with culture medium containing homogeneously distributed CCL26 (**b**). Each graph displays one representative experiment, containing migration data of at least N = 20 cells. T1, time point < 5 min; T2, time point = 2.5 h; T3, time point = 5 h; C, CCL26; H, hypoxia

Proteome profiling identified CCL26 as a cytokine whose secretion was almost completely abolished by hypoxia. To analyze the possible effects of CCL26 on the migratory behavior of monocytes, recombinant CCL26 was added into the migration chamber (Fig. 1) to generate a concentration gradient of this cytokine. The formation of a CCL26 gradient throughout the chamber resulted in a significant movement of monocytes away from the source of CCL26 and towards lower concentrations of the molecule, confirming chemorepulsive effects of CCL26 on monocytes (p < 0.05 Rayleigh test, Fig. 4a). Applying homogeneously distributed CCL26 to monocytes in a migration chamber with oxygen gradient abolished the described directed migration of monocytes towards the source of hypoxia (p > 0.05 Rayleigh test, Fig. 4b). A graphical presentation of the outcome of each independently performed experiment is displayed in Additional file 1: Figure S3.

## Discussion

Migration and accumulation of monocytes in hypoxic areas are features of several diseases and important determinants of tissue injury in chronic infections (e.g. rheumatic arthritis, arteriosclerosis) or ischemic diseases (e.g. myocardial and cerebral infarction) [5, 6]. Besides these disease-associated detrimental effects, there are also several reports suggesting monocytes to be involved in tissue repair, neoangiogenesis, and immunomodulation [18, 19]. Therefore, a deeper understanding of the underlying mechanism of hypoxia on monocyte recruitment and migration could enhance the development of novel treatment strategies for conditions and diseases associated with the infiltration of monocytes. Due to the involvement and complex interaction of various cell types and tissue-derived factors during inflammatory processes, the exact mechanisms of hypoxia-directed migration of monocytes are not fully understood yet. In this context, cell culture systems, resembling the in-vivo situation by employing stable oxygen gradients, could help to decipher the mechanisms involved in hypoxia-induced migration of monocytes.

So far, the generation of stable and precise oxygen gradients in cell cultures is mainly limited to the use of special microfluidic devices. These systems are capable of generating oxygen gradients for cell culture experiments using the principle of oxygen diffusion, oxygen scavenging by chemicals, or electrolytic reactions [20]. However, the mentioned systems are rather complex as they require microfabrication techniques, flow-producing equipment, or the use of supplemental gas tanks to generate a fast and stable oxygen gradient [20, 21]. In the presented study, we combined a commercially available, diffusion-based chemotaxis system with an established and well-described enzymatic hypoxia model that enables the generation of stable oxygen gradients for at least 6 h [14, 15, 17]. This hypoxia system represents a low-cost, easy to handle, and space-saving application, which can be easily utilized by other researchers to study migratory behavior of various cell types under defined oxygen gradients.

Until now, the role of molecular oxygen as chemoattractant or chemorepellent for human cells is only rudimentary investigated and the underlying cellular, as well as molecular mechanisms remain unclear. Nevertheless, several authors have proven the role of oxygen as a chemotactic factor, primarily for tumor cells. Interestingly, depending on the cell line employed, both the migration of tumor cells towards regions of higher oxygen levels as well as the attraction of cells to hypoxic areas have been reported [22–25]. To the best of our knowledge, our study demonstrated for the first time that hypoxia itself acts as a chemoattractant for primary human monocytes. In our in-vitro experiments, monocytes migrate along oxygen gradients toward regions of lower oxygen concentrations, resembling at least partially the in-vivo situation, where monocytes/macrophages migrate and accumulate within hypoxic tissues [3, 11].

**Fig. 5:**
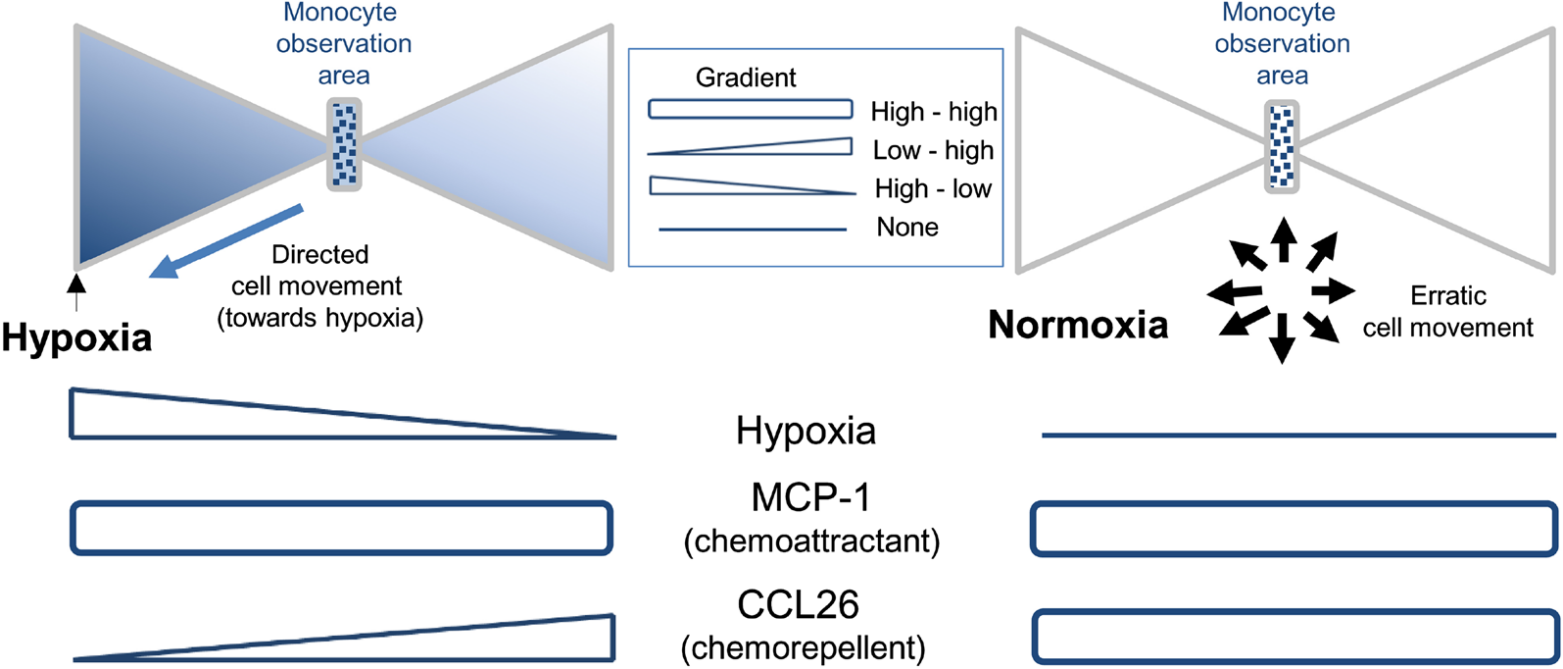
Hypothetical scheme explaining the possible relationship between hypoxia, MCP-1 levels, CCL26 gradients and directed migration of monocytes

Cytokines are low molecular weight proteins that are secreted by various cell types either constitutively or upon inflammatory stimuli and regulate complex physiological processes such as leukocyte recruitment and activation [3, 26]. Genomic sequencing of monocytes under hypoxic conditions revealed a dynamic change of the cytokine gene expression profile [3, 27]. Bosco et al. showed that the exposure of monocytes to hypoxia for 24 h resulted in an upregulation of several neutrophil attracting chemokines like CXCL2, CXCL3, CXCL5, CXCL6, and CXCL8, whereas the gene expression of chemokines that act mainly on monocytes/macrophages (e.g. MCP-1, CCL8, CCL15, CCL18, CCL19, CCL23, and CXCL11) was inhibited by hypoxia [27]. Our analysis of the cytokine/chemokine protein secretion pattern of monocytes revealed high secretion levels of chemokines responsible for monocyte migration like MCP-1, MIP-1α, and CCL5 under hypoxic as well as normoxic conditions. Interestingly, in our experiments, the exposure of monocytes to hypoxia was associated with a drastic attenuation of the secretion of several cytokines (e.g. CCL26, CCL1, CX3CL1, CCL17, and XCL1). The observation that monocytes migrate towards hypoxia and that this process is accompanied by a reduction of the auto- and/or paracrine release of cytokines, suggests that in addition to direct effects of hypoxia, a reduced secretion of potentially chemorepulsive chemokines under hypoxia could also be responsible for the directed monocyte migration. This hypothesis is supported by the fact that CCL26, the chemokine whose secretion was almost completely abolished by hypoxia, represents the only cytokine reported to possess chemorepulsive properties on monocytes. Ogilvie and colleagues identified CCL26 as a natural antagonist of CCR2, the main receptor for monocyte chemoattractant protein-1 (MCP-1), which regulates migration and infiltration of monocytes [28, 29]. In our setting, MCP-1 secretion was not regulated by hypoxia and high levels of MCP-1 were detected under normoxic as well as under hypoxic conditions. Ogilvie et al. showed that CCL26 can inhibit the MCP-1 induced migration of monocytes and is also capable of acting as a chemorepulsive molecule for monocytes itself [29]. The observed hypoxia-mediated reduction of chemorepulsive CCL26 molecules might directly result in monocyte migration towards hypoxia. Also, the lower levels of CCL26 observed under hypoxia could result in a reduced inhibition of CCR2 and therefore increase the chemoattractive effects of MCP-1. (Fig. 5). Concerning possible factors interacting with CCL26, preliminary dual immunofluorescent stainings of monocytes subjected to hypoxic gradients revealed colocalization of CCL26 with the advanced glycosylation end product (AGE) receptor (RAGE: Additional file 1: Figure S4) which has been associated with activation and migration of monocytes [30–35]. It should be noted that these preliminary findings do not provide evidence of a causal involvement of RAGE in hypoxia/CCL26-mediated monocyte migration and further studies are necessary to clarify the relevance of RAGE for the described mechanisms.

## Conclusion

Hypoxia alone is sufficient to induce directed migration of human monocytes. CCL26 seems to be a key player in hypoxia-mediated monocyte migration and may represent a clinical target for modulating monocyte migration in ischemia associated illnesses.

## Supplementary information

**Additional file 1: Figure S1.** Red dots indicate the spatial representation of the mean endpoint after the evaluation of cell movements of at least 20 cells per experiment (N = 4–5). A: oxygen gradient (hypoxia); B: no oxygen gradient (normoxia). P-values were calculated using circular statistics (Rayleigh test). Black lines inside the quadrants show the linear correlation of the center of mass in each experimental group. **Figure S2.** Detailed description of the array proteins. **Figure S3.** Red dots indicate the spatial representation of the mean endpoint after the evaluation of cell movements of at least 20 cells per experiment (N = 3). A: no oxygen gradient (normoxia) in combination with a gradient of CCL26; B: oxygen gradient (hypoxia) with homogeneously distributed CCL26. P-values were calculated using circular statistics (Rayleigh test). Black lines inside the quadrants show the linear correlation of the center of mass in each experimental group. **Figure S4.** Upper panel: Detection of RAGE by Western blotting in cell lysates of monocytes subjected to hypoxic and normoxic conditions (3 representative samples out of N = 7 are shown). Columns show the mean of N = 7 experiments, bars denote SD. Exp, experiment; H, hypoxia, N, normoxia. Lower panel: Colocalization of RAGE and CCL26 in monocytes (3 representative samples out of N = 7 are shown). Yellow color is indicative of cellular RAGE/CCL26 colocalization. Scale bars represent 3 µm. Exp, experiment.

## Data Availability

The datasets used and/or analyzed during this study are available from the corresponding author on reasonable request.
