# Supplementary material for: Hypoxia directed migration of human naïve monocytes is associated with an attenuation of cytokine release: indications for a key role of CCL26
Source: J Transl Med. 2020 Oct 21;18:404. doi: 10.1186/s12967-020-02567-7 (PMC7579884; doi:10.1186/s12967-020-02567-7)
Supplement: Supplementary file 1 — Additional file 1: Figure S1. Red dots indicate the spatial representation of the mean endpoint after the evaluation of cell movements of at least 20 cells per experiment (N = 4–5). A: oxygen gradient (hypoxia); B: no oxygen gradient (normoxia). P-values were calculated using circular statistics (Rayleigh test). Black lines inside the quadrants show the linear correlation of the center of mass in each experimental group. Figure S2. Detailed description of the array proteins. Figure S3. Red dots indicate the spatial representation of the mean endpoint after the evaluation of cell movements of at least 20 cells per experiment (N = 3). A: no oxygen gradient (normoxia) in combination with a gradient of CCL26; B: oxygen gradient (hypoxia) with homogeneously distributed CCL26. P-values were calculated using circular statistics (Rayleigh test). Black lines inside the quadrants show the linear correlation of the center of mass in each experimental group. Figure S4. Upper panel: Detection of RAGE by Western blotting in cell lysates of monocytes subjected to hypoxic and normoxic conditions (3 representative samples out of N = 7 are shown). Columns show the mean of N = 7 experiments, bars denote SD. Exp, experiment; H, hypoxia, N, normoxia. Lower panel: Colocalization of RAGE and CCL26 in monocytes (3 representative samples out of N = 7 are shown). Yellow color is indicative of cellular RAGE/CCL26 colocalization. Scale bars represent 3 µm. Exp, experiment. [file 12967_2020_2567_MOESM1_ESM.docx]

*
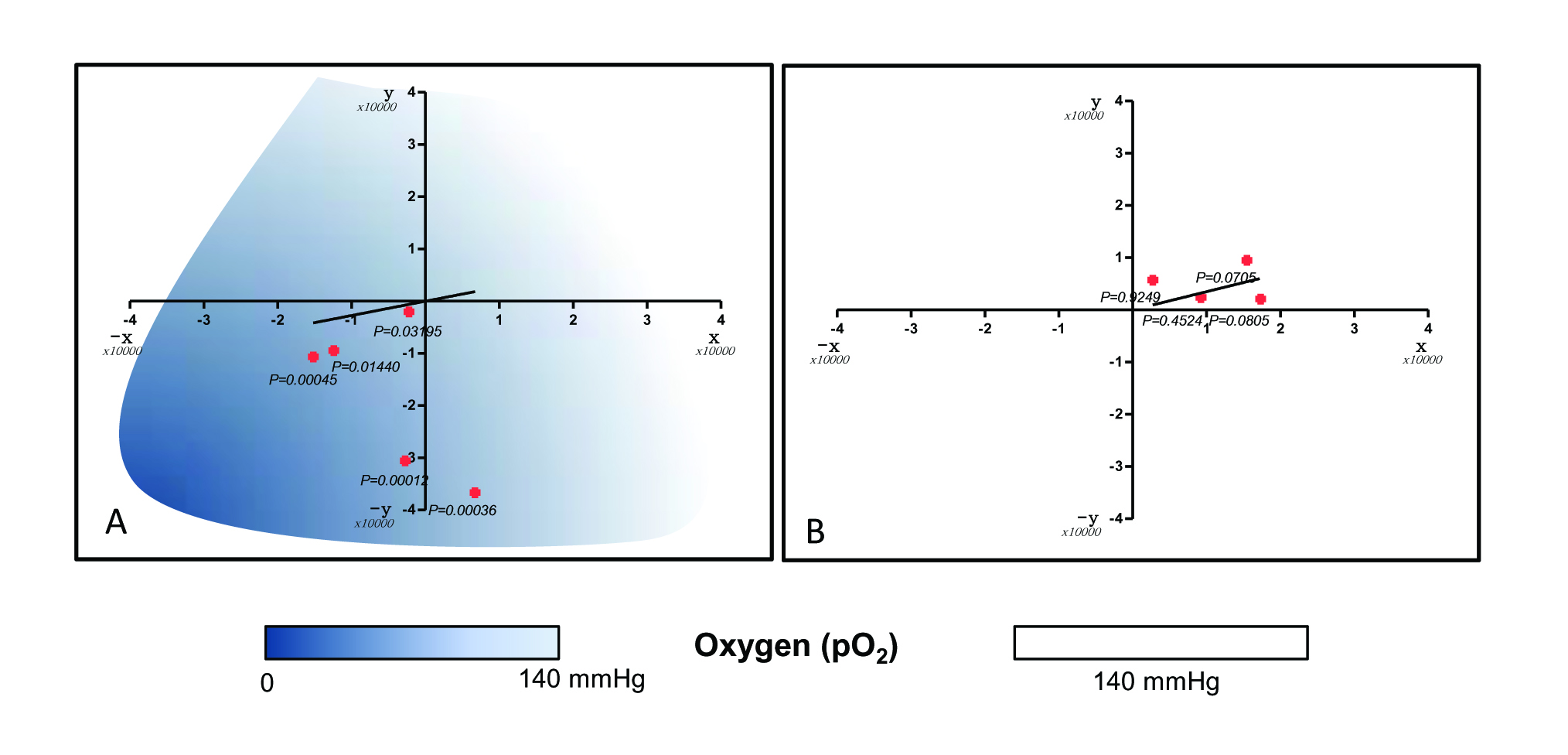
*

*Figure S1: Red dots indicate the spatial representation of the mean endpoint after the evaluation of cell movements of at least 20 cells per experiment (N=4-5). A: oxygen gradient (hypoxia); B: no oxygen gradient (normoxia). P-values were calculated using Circular statistics (Rayleigh test). Black lines inside the quadrants show the linear correlation of the center of mass in each experimental group.*


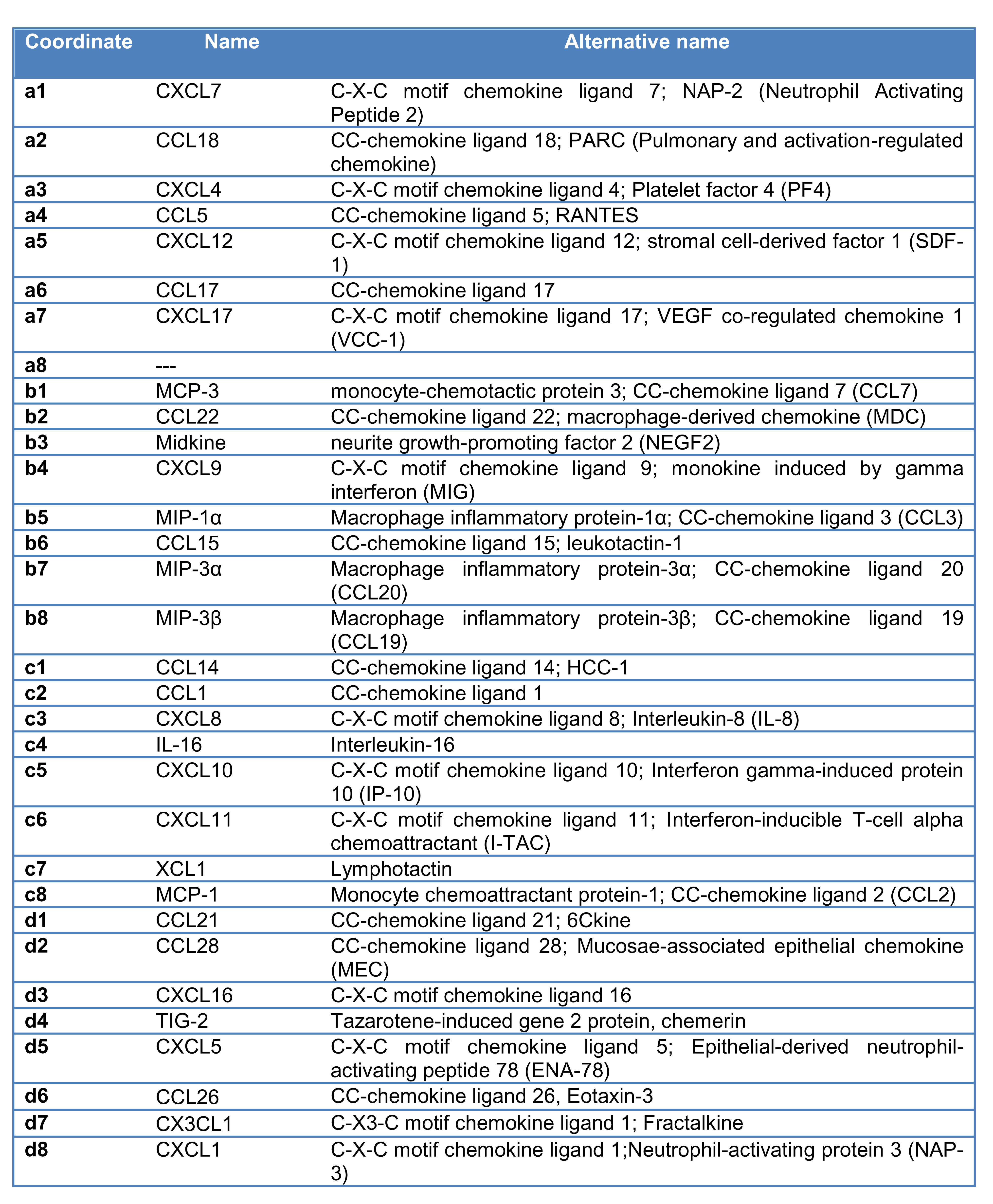


*Figure S2: Detailed description of the array proteins.*

*
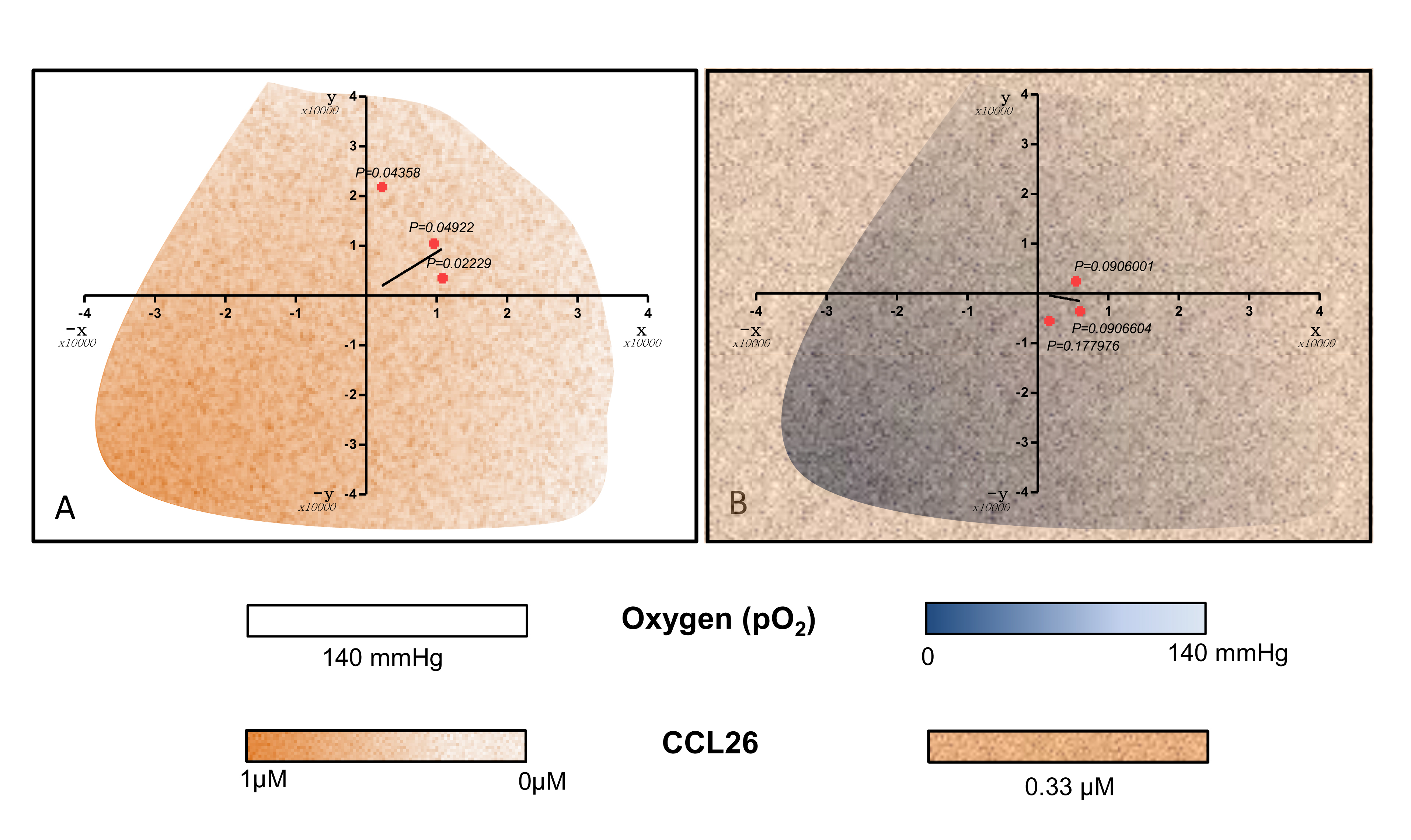
Figure S3: Red dots indicate the spatial representation of the mean endpoint after the evaluation of cell movements of at least 20 cells per experiment (N=3). A: no oxygen gradient (normoxia) in combination with a gradient of CCL26; B: oxygen gradient (hypoxia) with homogeneously distributed CCL26. P-values were calculated using Circular statistics (Rayleigh test). Black lines inside the quadrants show the linear correlation of the center of mass in each experimental group.*

*
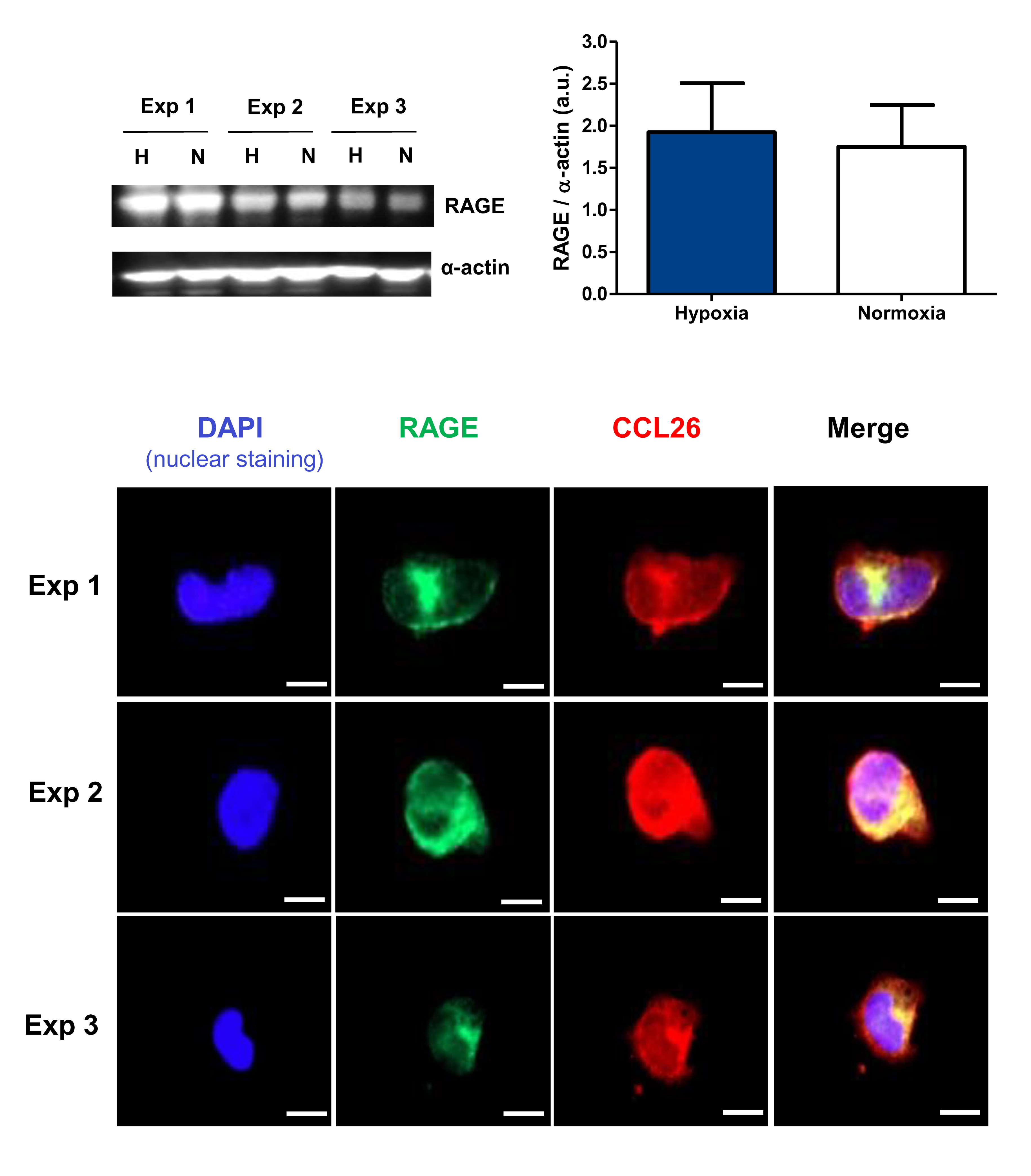
*

*Figure S4: Upper panel: Detection of RAGE by Western blotting in cell lysates of monocytes subjected to hypoxic and normoxic conditions (3 representative samples out of N=7 are shown). Columns show the mean of N=7 experiments, bars denote SD. Exp, experiment; H, hypoxia, N, normoxia. Lower panel: Colocalization of RAGE and CCL26 in monocytes (3 representative samples out of N=7 are shown). Yellow color is indicative of cellular RAGE/CCL26 colocalization. Scale bars represent 3µm. Exp, experiment.*
